# Confirmation of Pinnatoxins and Spirolides in Shellfish and Passive Samplers from Catalonia (Spain) by Liquid Chromatography Coupled with Triple Quadrupole and High-Resolution Hybrid Tandem Mass Spectrometry

**DOI:** 10.3390/md12063706

**Published:** 2014-06-23

**Authors:** María García-Altares, Alexis Casanova, Vaishali Bane, Jorge Diogène, Ambrose Furey, Pablo de la Iglesia

**Affiliations:** 1 Institute of Agrifood Research and Technology (IRTA), Poble Nou Road, km. 5.5, Sant Carles de la Ràpita 43540, Spain; E-Mails: maria.garciaaltares@irta.cat (M.G.-A.); alexis.casanova@irta.cat (A.C.); jorge.diogene@irta.cat (J.D.); 2 Mass Spectrometry Research Centre (MSRC) and PROTEOBIO Research Group, Department of Chemistry, Cork Institute of Technology, Bishopstown, Cork, Ireland; E-Mails: vaishali.p.bane@mycit.ie (V.B.); ambrose.furey@cit.ie (A.F.)

**Keywords:** spirolides, pinnatoxins, Mediterranean Sea, triple quadrupole mass spectrometry, high resolution mass spectrometry, orbitrap FT-MS

## Abstract

Cyclic imines are lipophilic marine toxins that bioaccumulate in seafood. Their structure comprises a cyclic-imino moiety, responsible for acute neurotoxicity in mice. Cyclic imines have not been linked yet to human poisonings and are not regulated in Europe, although the European Food Safety Authority requires more data to perform a conclusive risk assessment for consumers. This work presents the first detection of pinnatoxin G (PnTX-G) in Spain and 13-desmethyl spirolide C (SPX-1) in shellfish from Catalonia (Spain, NW Mediterranean Sea). Cyclic imines were found at low concentrations (2 to 60 µg/kg) in 13 samples of mussels and oysters (22 samples analyzed). Pinnatoxin G has been also detected in 17 seawater samples (out of 34) using solid phase adsorption toxin tracking devices (0.3 to 0.9 µg/kg-resin). Pinnatoxin G and SPX-1 were confirmed with both low and high resolution (<2 ppm) mass spectrometry by comparison of the response with that from reference standards. For other analogs without reference standards, we applied a strategy combining low resolution MS with a triple quadrupole mass analyzer for a fast and reliable screening, and high resolution MS LTQ Orbitrap^®^ for unambiguous confirmation. The advantages and limitations of using high resolution MS without reference standards were discussed.

## 1. Introduction

**Figure 1:**
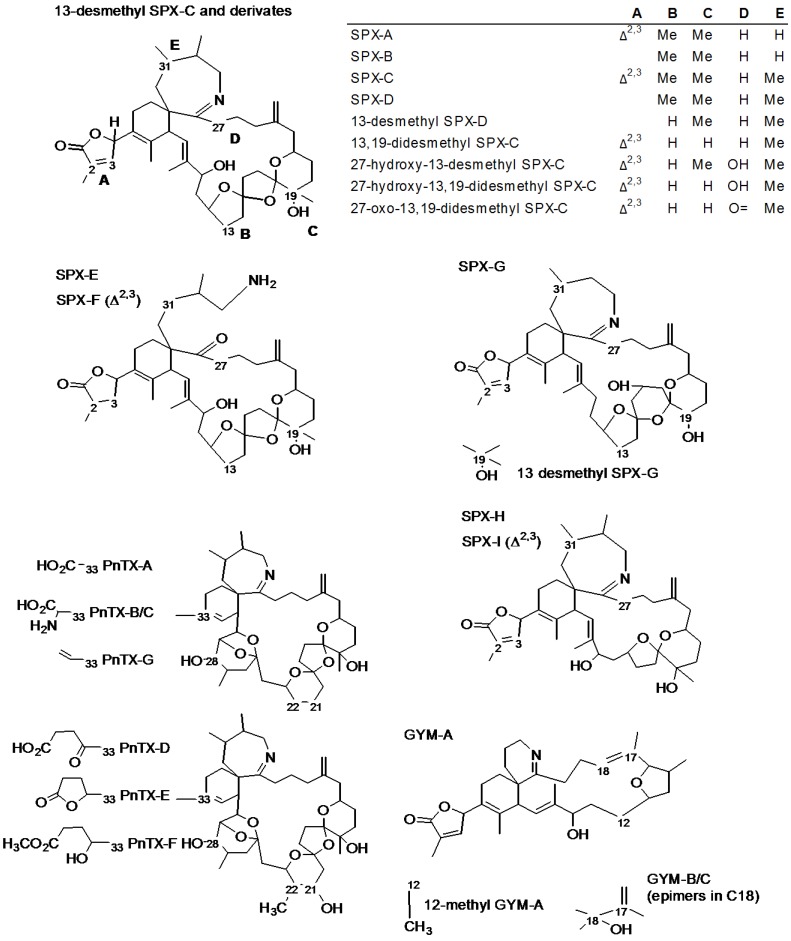
Structures of spirolides (13-desmethyl SPX-C type, SPX-E type, SPX-G type and SPX-H type), pinnatoxins (PnTX-A type and PnTX-D type) and gymnodimines (GYM-A type).

The group of emerging toxins called cyclic imines includes eight types of compounds produced by marine dinoflagelates: spirolides (SPXs, 13 desmethyl SPX-C, also known as SPX-1, being the reference toxin for this group), pinnatoxins (PnTXs, reference compound PnTX-G), gymnodimines (GYMs, reference compound GYM-A), pteriatoxins (PtTXs), prorocentrolides, spiro-prorocentrimine [1,2], symbioimines [3] and portimine [4]. They all share a cyclic imine group, which is extremely rare, that works as the pharmacophore [1,2], as confirmed for the 6,6-spiroimine in GYM-A [5]. Spirolides and pinnatoxins comprise more than twenty different analogs (Table 1) with a similar structure (Figure 1) characterized by the 6,7-spiro ring with an imine group and a spiro-linked polyether moiety [6].

**Table 1:** List of spirolide and pinnatoxin analogs reported to date (reference compound of each group in italics). Retention times (RT) and relative retention times in brackets, exact mass and elemental formula of the molecular ions and diagnostic fragments. Relative retention times were calculated from literature by approximation from a chromatogram (a) or by calculation from retention time data (b). The mass of the electron is 1/1837 amu.

Toxin	RT (min)	[M + H]^+^		Fragments	
		Exact Mass (*m/z*)	Formula	Exact Mass (*m/z*)	Formula
13-desmethyl Spirolide C [7,8] (SPX-1)	1.00	692.4521	C_42_H_62_NO_7_	674.4415	C_42_H_60_NO_6_
				444.3108	C_27_H_42_NO_4_
				164.1434	C_11_H_18_N
Spirolide A [7]	(0.90) a	692.4521	C_42_H_62_NO_7_	150.1277	C_10_H_16_N
Spirolide B [6,7]	(1.00) a	694.4677	C_42_H_64_NO_7_	150.1278	C_10_H_16_N
Spirolide C [7]	(1.05) a	706.4677	C_43_H_64_NO_7_	458.3265	C_28_H_44_NO_4_
				164.1434	C_11_H_18_N
Spirolide D [6]	(1.10) a	708.4834	C_43_H_66_NO_7_	608.4310	C_38_H_58_NO_5_
				458.3265	C_28_H_44_NO_4_
				164.1434	C_11_H_18_N
Spirolide E [9]	(1.30) a	710.4637	C_42_H_64_NO_8_	444.3108	C_27_H_42_NO_4_
Spirolide F [9]		712.4794	C_42_H_66_NO_8_	444.3108	C_27_H_42_NO_4_
Spirolide G [10]	(1.60) a	692.4521	C_42_H_62_NO_7_	378.2639	C_22_H_36_NO_4_
				164.1434	C_11_H_18_N
Spirolide H [11]	Not reported	650.4415	C_40_H_60_NO_6_	402.3003	C_25_H_40_NO_3_
				164.1422	C_11_H_18_N
Spirolide I [11]	Not reported	652.4572	C_40_H_62_NO_6_	402.3003	C_25_H_40_NO_3_
				164.1434	C_11_H_18_N
13-desmethyl Spirolide D [12,13]	(1.06) a	694.4677	C_42_H_64_NO_7_	676.4572	C_42_H_62_NO_6_
				444.3108	C_27_H_42_NO_4_
				164.1434	C_11_H_18_N
13,19-didesmethyl Spirolide C [10,12]	(0.94) b	678.4364	C_41_H_60_NO_7_	430.2952	C_26_H_40_NO_4_
				164.1434	C_11_H_18_N
27-hydroxy-13,19-didesmethyl Spirolide C [12,14]	(0.91) b	694.4313	C_41_H_60_NO_8_	464.3007	C_26_H_42_NO_6_
				180.1383	C_11_H_18_NO
27-hydroxy-13-desmethyl Spirolide C [14]	(0.92) b	708.4470	C_42_H_62_NO_8_	478.3163	C_27_H_44_NO_6_
				180.1383	C_11_H_18_NO
27-oxo-13,19-didesmethyl Spirolide C [14]	(0.99) b	692.4157	C_41_H_58_NO_8_	444.2745	C_26_H_38_NO_5_
				178.1226	C_11_H_16_NO
20-methyl Spirolide G [15,16]	(1.06) b	706.4677	C_43_H_64_NO_7_	392.2795	C_23_H_38_NO_4_
				164.1434	C_11_H_18_N
*Pinnatoxin G* [17]	1.00	694.4677	C_42_H_64_NO_7_	676.4572	C_42_H_62_NO_6_
				458.3265	C_28_H_44_NO_4_
				164.1434	C_11_H_18_N
Pinnatoxin A [17,18,19]	(0.77) a	712.4419	C_41_H_62_NO_9_	458.3265	C_28_H_44_NO_4_
				164.1434	C_11_H_18_N
Pinnatoxin B/C (epimers) [19,20]	Not reported	741.4685	C_42_H_65_N_2_O_9_	458.3265	C_28_H_44_NO_4_
				164.1434	C_11_H_18_N
Pinnatoxin D [17]	Not reported	782.4838	C_45_H_68_NO_10_	446.3265	C_27_H_44_NO_4_
				164.1434	C_11_H_18_N
Pinnatoxin E [17]	(0.83) a	784.4994	C_45_H_70_NO_10_	446.3265	C_27_H_44_NO_4_
				164.1434	C_11_H_18_N
Pinnatoxin F [17]	(0.97) a	766.4889	C_45_H_68_NO_9_	446.3265	C_27_H_44_NO_4_
				164.1434	C_11_H_18_N

Cyclic imines display a fast acute neurotoxicity in mice (by intraperitoneal injection) with an all-or-nothing response (they cause death in less than 20 min, or no symptoms at all). They affect the central nervous system as a very potent and non-selective antagonist of nicotinic receptors [1,2]. The mechanism of action is the same for all studied cyclic imines [1,2,21] and both GYM-A and SPX-1 show high affinity and slow dissociation of the bound ligand to their receptors [22,23]. Nevertheless, not all cyclic imines are equally potent: SPX-1 showed about 300 fold more activity than GYM-A on equimolar basis in a *in vivo* study about neuromuscular excitability in mice [24]. To date, there is still a lack of information on the chronic toxicity of cyclic imines.

Three genetically distant species of dinoflagelates produce structurally related cyclic imines: *Vulcanodinium rugosum* produces PnTXs [25] and portimine [4], *Karenia selliformis* produces GYMs [26], and *Alexandrium ostenfeldii* (conspecific of *A. peruvianum* [27]) produce SPXs [28,29]. Interestingly, the novel 12-methyl GYM-A [30] was found in an *A. ostenfeldii* strain also produced SPXs [31].

Pinnatoxins were isolated for the first time from shellfish samples in Japan [32] in 1990 and later reported in Australia and New Zealand, Canada and Europe [16,25,33,34]. Spirolides were detected for the first time in 1995 in Canada [6,9,28]. As explained in the last two comprehensive reviews on cyclic imines [1,2], SPXs have been found in shellfish, plankton, and sea water samples in different parts of the world [9,29,35,36,37,38,39]. In 2006, SPX-1 was detected and quantified (13 to 20 µg/kg) for the first time on the Atlantic coast of Spain, in samples from shellfish harvesting areas in the NW of the country [40].

The presence of cyclic imines in the Mediterranean Sea was confirmed in 2002, when GYM-A was found for the first time in clams from Tunisia [41]. Spirolides were reported for the first time in the Mediterranean area in 2007 in mussels from the Italian coasts of the Northern Adriatic Sea, where they were the main contributor to toxicity and shown a very complex profile, including 13-desmethyl SPX-C, 13,19-didesmethyl SPX-C, 27-hydroxy-13,19-didesmethyl SPX-C (unreported until then) and several new minor spirolides from which two of them (27-hydroxy-13-desmethyl SPX-C and 27-oxo-13,19-didesmethyl SPX-C) where isolated and structurally described [12,14,42]. In 2012, very high concentrations of PnTX-G and traces of PnTX-A were found, in mussels and clams from Ingril lagoon, in the SE of France (up to 600 μg PnTX-G /kg of shellfish tissue and 4.7 pg/cell of *V. rugosum*) [34]. The recent discovery of cyst of *V. rugosum* in Fangar Bay (Catalonia, Spain) suggested that PnTXs might be also present in our study area [43].

Cyclic imines have not been directly linked to human intoxications, but the European Food Safety Authority requested more exposure data to properly assess the risk that cyclic imines pose to shellfish consumers [44]. This paper shows the results of a preliminary survey conducted in Catalonia (Spain), NW Mediterranean Sea, to assess the presence of cyclic imines in shellfish and seawater samples from solid phase adsorption toxin tracking (SPATT) devices. Our aim was to develop a strategy to detect trace amounts of cyclic imines in marine samples guaranteeing their correct identification. Thus, we discuss the complementation of two liquid chromatography coupled with mass spectrometry (LC-MS) techniques (low and high resolution MS (HRMS)) to quickly screen the samples, unambiguously confirm the presence of cyclic imines, quantify them and ensuring one avoids false positives.

## 2. Results and Discussion

Two cyclic imines, PnTX-G and SPX-1, were found at low concentrations (2 to 60 μg PnTX-G/kg and 2 to 16 μg SPX-1/kg) in 13 samples of mussels and oysters (out of 22 samples analyzed, Table 2). Pinnatoxin G has also been detected in 17 seawater samples (out of 34) using solid phase adsorption toxin tracking devices (SPATT, 0.3 to 0.9 μg/kg-resin, Table 3). The quality assessment of the methods of quantification and confirmation was satisfactory. We looked for other cyclic imines (Table 1) and acyl ester derivatives of cyclic imines (Section 2.3), but we could not confirm their presence in the samples.

**Table 2:** Confirmation with LTQ Orbitrap Discovery^®^ FT-MS and quantification with 3200QTrap^®^ of SPX-1 and PnTX-G in shellfish. ND: Not detected; NC: Not confirmed; NQ: Not quantified. Δ ppm: mass error in ppm; [M + H]^+^: Molecular Ion (SPX-1: 692.4521 *m/z*; PnTX-G: 694.4677 *m/z*); Fragment 1: 164.1434 *m/z* (both SPX-1 and PnTX-G); Fragment 2: 444.3108 (SPX-1) and 458.3265 *m/z* (PnX-G); Fragment 3: 674.4415 *m/z* (SPX-1) and 676.4572 *m/z* (PnTX-G).

Sample Details	SPX-1 (Δ ppm)		SPX-1 (μg/kg)	PnTX-G (Δ ppm)		PnTX-G (μg/kg)
OYS110102	[M + H]^+^	1.0		[M + H]^+^	Δppm > 5 ppm	
Oyster	Frag 1	−0.2	3.6 ± 0.1	Frag 1	Δppm > 5 ppm	ND
January 2011	Frag 2	1.2		Frag 2	Δppm > 5 ppm	
Fangar Bay	Frag 3	−2.3		Frag 3	Δppm > 5 ppm	
OYS110103	[M + H]^+^	−0.2		[M + H]^+^	Δppm > 5 ppm	
Oyster	Frag 1	0.2	3.5 ± 0.8	Frag 1	Δppm > 5 ppm	ND
January 2011	Frag 2	−0.9		Frag 2	Δppm > 5 ppm	
Fangar Bay	Frag 3	−3.5		Frag 3	Δppm > 5 ppm	
OYS110105	[M + H]^+^	5.0		[M + H]^+^	40.6	
Mussel	Frag 1	−0.2	2.2 ± 0.6	Frag 1	−0.2	3.8 ± 0.6
January 2011	Frag 2	0.5		Frag 2	−2.6	
Fangar Bay	Frag 3	0.4		Frag 3	−0.7	
OYS110115	[M + H]^+^	0.4		[M + H]^+^	Δppm > 5 ppm	
Oyster	Frag 1	−0.2	5 ± 1	Frag 1	Δppm > 5 ppm	ND
January 2011	Frag 2	0.3		Frag 2	Δppm > 5 ppm	
Fangar Bay	Frag 3	2.3		Frag 3	Δppm > 5 ppm	
MUS110116	[M + H]^+^	1.6		[M + H]^+^	1.3	
Mussel	Frag 1	0.1	3.6 ± 0.18	Frag 1	0.5	3 ± 1
January 2011	Frag 2	−0.1		Frag 2	−0.2	
Fangar Bay	Frag 3	0.9		Frag 3	−0.1	
OYS110117	[M + H]^+^	−0.1		[M + H]^+^	Δppm > 5 ppm	
Oyster	Frag 1	0.1	6.6 ± 0.7	Frag 1	Δppm > 5 ppm	ND
January 2011	Frag 2	−0.1		Frag 2	Δppm > 5 ppm	
Fangar Bay	Frag 3	−0.9		Frag 3	Δppm > 5 ppm	
OYS110205	[M + H]^+^	0.4		[M + H]^+^	Δppm > 5 ppm	
Oyster	Frag 1	−0.1	5.8 ± 0.5	Frag 1	Δppm > 5 ppm	ND
February 2011	Frag 2	0.1		Frag 2	Δppm > 5 ppm	
Fangar	Frag 3	1.6		Frag 3	Δppm > 5 ppm	
MUS110205	[M + H]^+^	−0.2		[M + H]^+^	2.2	
Mussel	Frag 1	0.3	NQ	Frag 1	0.5	4.1 ± 0.1
February 2011	Frag 2	−0.2		Frag 2	−1.8	
Fangar Bay	Frag 3	0.9		Frag 3	0.4	
MUS1027	[M + H]^+^	0.2		[M + H]^+^	42.6	
Mussel	Frag 1	−0.6	3 ± 2	Frag 1	−0.5	2.2 ± 0.1
February 2011	Frag 2	−1.0		Frag 2	−1.9	
Fangar Bay	Frag 3	0.3		Frag 3	−2.00	
OYS110208	[M + H]^+^	0.5		[M + H]^+^	Δppm > 5 ppm	
Oyster	Frag 1	−0.2	4 ± 1	Frag 1	Δppm > 5 ppm	ND
February 2011	Frag 2	0.1		Frag 2	Δppm > 5 ppm	
Fangar	Frag 3	−1.0		Frag 3	Δppm > 5 ppm	
MUS120520	[M + H]^+^	1.0		[M + H]^+^	−1.1	
Mussel	Frag 1	−0.5	NC	Frag 1	−0.5	39 ± 6
May 2012	Frag 2	Δppm > 5 ppm		Frag 2	−0.7	
Fangar Bay	Frag 3	Δppm > 5 ppm		Frag 3	−0.9	
MUS120523	[M + H]^+^	−2.1		[M + H]^+^	0.2	
Mussel	Frag 1	−0.1	16 ± 1	Frag 1	−0.1	59 ± 5
May 2012	Frag 2	0.4		Frag 2	−0.1	
Retail market	Frag 3	−0.1		Frag 3	−0.1	
MUS120524	[M + H]^+^	Δppm > 5 ppm		[M + H]^+^	−1.1	
Mussel	Frag 1	−1.1	NC	Frag 1	−0.5	58 ± 2
May 2012	Frag 2	Δppm > 5 ppm		Frag 2	−0.9	
Fangar Bay	Frag 3	Δppm > 5 ppm		Frag 3	−0.9	

**Table 3:** Confirmation with LTQ Orbitrap Discovery^®^ FTMS and quantification with 3200QTrap^®^ of PnTX-G in SPATT samples from Alfacs Bay. Δ ppm: mass error in ppm; [M + H]^+^: Molecular Ion (PnTX-G: 694.4677 *m/z*); Fragment 1: 164.1434 *m/z*; Fragment 2: 458.3265; Fragment 3: 676.4572 *m/z*.

Sample Name and Date	PnTX-G (Δppm)		PnTX-G (μg/kg)
TB_1028 February 2007	[M + H]^+^	0.9	
	Frag 1	0.3	0.47 ± 0.01
	Frag 2	−0.8	
	Frag 3	−0.1	
TB_1029 February 2007	[M + H]^+^	1.3	
	Frag 1	0.6	0.65 ± 0.09
	Frag 2	−0.8	
	Frag 3	−0.1	
TB_1036 February 2007	[M + H]^+^	−1.1	
	Frag 1	0.9	0.70 ± 0.05
	Frag 2	−0.1	
	Frag 3	0.4	
TB_1037 February 2007	[M + H]^+^	2.0	
	Frag 1	−3.3	0.75 ± 0.03
	Frag 2	2.6	
	Frag 3	3.5	
TB_1359 January 2008	[M + H]^+^	1.9	
	Frag 1	−2.6	0.48 ± 0.03
	Frag 2	−3.8	
	Frag 3	−3.1	
TB_1360 January 2008	[M + H]^+^	3.8	
	Frag 1	−2.5	0.28 ± 0.02
	Frag 2	−3.7	
	Frag 3	−2.9	
TB_1363 January 2008	[M + H]^+^	1.9	
	Frag 1	−2.7	0.93 ± 0.02
	Frag 2	−3.9	
	Frag 3	−3.2	
TB_1367 January 2008	[M + H]^+^	4.1	
	Frag 1	−3.4	0.65 ± 0.06
	Frag 2	−4.7	
	Frag 3	−4.0	
TB_1374 July 2008	[M + H]^+^	−0.1	
	Frag 1	−3.4	0.55 ± 0.01
	Frag 2	−4.3	
	Frag 3	−3.4	
TB_1378 July 2008	[M + H]^+^	−2.8	
	Frag 1	−3.3	0.47 ± 0.07
	Frag 2	−4.1	
	Frag 3	−3.4	
TB_1379 July 2008	[M + H]^+^	−1.1	
	Frag 1	−3.1	0.34 ± 0.02
	Frag 2	−4.8	
	Frag 3	−2.5	
TB_1381 July 2008	[M + H]^+^	8.7	
	Frag 1	−3.3	0.33 ± 0.02
	Frag 2	−5.0	
	Frag 3	−4.0	
TB_1614 Juny 2009	[M + H]^+^	−0.8	
	Frag 1	−2.9	0.58 ± 0.06
	Frag 2	−5.1	
	Frag 3	−1.0	

### 2.1. Unambiguous Confirmation and Quantification of PnTX-G and SPX-1

**Figure 2:**
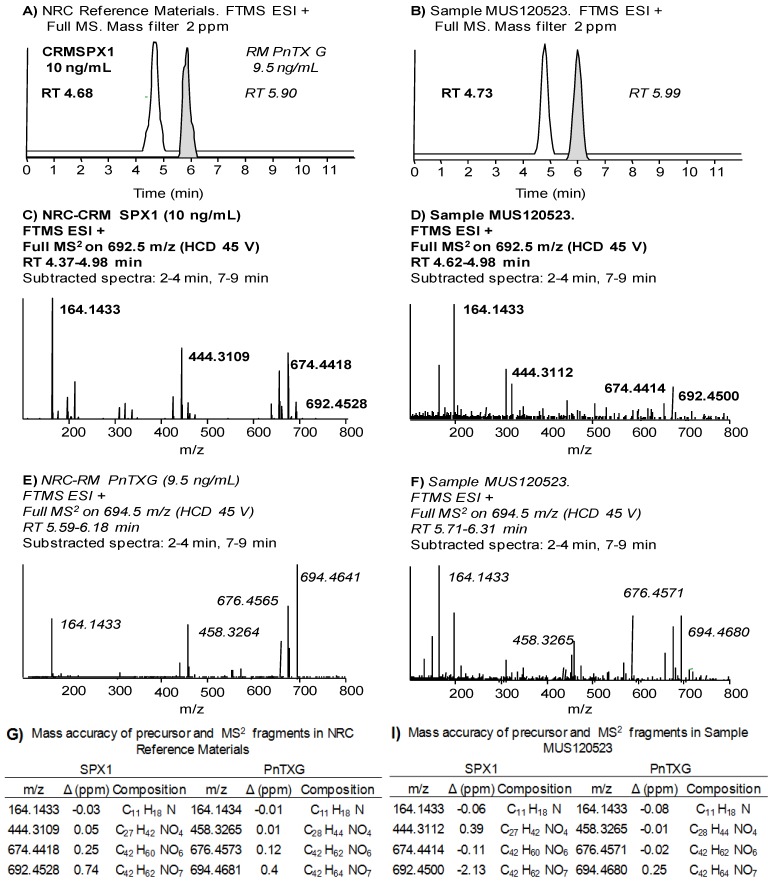
(**A**,**B**) Chromatograms; (**C**,**D**) MS^2^ product scans; and (**G**,**I**) assigned formulae and mass accuracy measurements (Δ in ppm) of the precursor ions and the diagnostic product ions of SPX-1 and PnTX-G in the reference standard materials (**A**,**C**,**G**) and sample MUS120523 (**B**,**D**,**I**).

Figure 2 illustrates the unambiguous confirmation of PnTX-G and SPX-1 in a mussel sample from Fangar Bay collected in May 2012 (MUS120523). Pinnatoxin-G and SPX-1 were confirmed by their retention time (RT) compared to that of the standards (<1% difference, Figure 2A,B), the mass accuracy of the precursor and of three diagnostic fragments (<2 ppm, Figure 2D,F,I) and one ion ratio. The final concentration in the sample was 60 ± 5 µg/kg PnTX-G and 16 ± 1 µg/kg SPX-1.

Following the same approach, we analyzed 22 samples of shellfish (14 mussels and 8 oysters, Table 2) that were initially screened by triple quadrupole LC-MS/MS: 4 mussel samples from Alfacs Bay (Catalonia, Spain), 8 from Fangar Bay (Catalonia, Spain) and 2 mussel samples from retail market, one oyster sample from Alfacs Bay, and 7 from Fangar Bay. We confirmed the presence of PnTX-G in 6 mussels from Fangar Bay and one mussel from the retail market, with concentrations from 2 to 60 μg/kg (Table 2). SPX-1 was confirmed in 5 mussel samples and 6 oyster samples from Fangar Bay, and in one mussel samples from retail market. The SPX-1 concentrations ranged from 2 to 16 μg/kg. In summary, 13 samples were confirmed to contain one cyclic imine (PnTX-G or SPX-1), and 4 mussel samples from Fangar Bay and one from the retail market had both toxins. Nine samples showed characteristic transitions for PnTX-G and/or SPX-1 in the triple quadrupole LC-MS/MS at very low intensity, but could not meet the confirmation requirements in the LTQ Orbitrap method. Separately, a total of 34 SPATT samples immersed for a week in the seawater of Alfacs Bay (Table 3) were selected and analyzed from the period 2006 to 2009. Pinnatoxin G was present in 17 SPATT samples from 2007, 2008 and 2009 and its concentration was quantified in 13 SPATT samples, ranging from 0.28 to 0.93 μg/kg resin.

Although several SPATT samples showed some of the diagnostic fragments of SPX-1 in the MS^2^ product scan of 692.5 *m/z*, the precursor ion for SPX-1 was not detected in the full MS scan in any SPATT sample using a 5 ppm mass window. This could be explained by the relative low abundance of the ion 692.4521 *m/z* in the presence of much more abundant ions present in the matrix. LTQ Orbitraps operate with a C-trap that storage the ions coming from the ion trap to be injected into the Orbitrap analyzer. The filling of the C-trap is controlled by the automatic gain control function of the instrument (based on a pre-scan in the ion trap that optimizes the number of ions stored in the C-trap [45]). In full MS experiments, the detection of low abundance ions in the presence of high abundance ions is therefore challenging for two reasons: (1) the space charge capacity of the C-trap may get saturated with ions present in high abundance and thus the sensitivity for low abundance ions gets limited; and (2) the high dynamic range of the ion population (the ratio of maximum signal to minimum signal) compromises the mass accuracy measurements of the ions with too low and too high intensity [46].

### 2.2. Study of PnTX-G and SPX-1 Analogs without Reference Standard Materials

Pinnatoxins and spirolides comprise a long list of more than 20 analogs (Table 1), most of them without available reference standard materials. High resolution mass spectrometry (HRMS) suits these applications: It enables the screening of an unlimited list of precursor ions in full scan mode, and provides great selectivity thanks to its high resolving power and precision in mass accuracy measurements. Thus, HRMS reduces the dependence on reference standard materials to identify marine biotoxins [47,48].

Two studies from 2011 explored the capabilities of the Orbitrap^®^ mass analyzer to screen marine toxins in shellfish samples, and in 2014 Domènech *et al.* [49] validated a quantitative method for lipophilic toxins. The screening methods were very comprehensive: Gerssen *et al.* [47] created an exportable search library for 85 lipophilic marine toxins, and Blay *et al.* [48] included the most common lipophilic and hydrophilic marine toxins in shellfish. However, the screening methods had different approaches using the same instrumentation (LC-HR Orbitrap FT-MS 100,000 resolution at 400 *m/z* at Full Width at Half Maximum, FWHM): While Blay *et al.* [48] relied only on the extraction of 5 ppm mass windows around the calculated mass of the precursor ions in full MS scans; Gerssen *et al.* [47] claimed that this approach is insufficient for confirmation, thus the study included criteria such as retention times of the analytes, spectral information, and blank samples to assess the potential false positives. Domènech *et al.* [49] also included those confirmation criteria, which could be potentially applied to real samples.

A suitable selection of the mass tolerance error is essential to provide optimum selectivity: the narrower the mass tolerance error, the less “false positives” (chromatographic peaks produced by compounds with an exact mass in the mass tolerance range). On the other hand, physical resolution and precision in mass accuracy measurements of the instrument determine the width of the mass error tolerance: setting a too narrow tolerance may lead to false negatives due to shifts in accurate mass measurements caused by hidden isobaric interferences and/or distortion of chromatographic peaks [50,51]. Defining an appropriate mass error tolerance seems to be a fit-for-purpose task [50], thus taking into account the resolution of our LTQ Orbitrap Discovery^®^ (resolution 30,000 at 400 *m/z* FWHM) and the good precision in mass accuracy measurements for PnTX-G and SPX-1 (<2 ppm, Section 2.4.2), we screened the samples for PnTXs and SPXs with a 10 ppm mass tolerance window. This tolerance widely accepted in HRMS applications led to a remarkable number of false positives, even in methanol blanks (see Supplementary Information). Therefore we investigated the isotopic patterns and MS^2^ full scans as additional confirmation tools.

Isotopic pattern and relative isotope abundance (RIA) can be used as confirmation criteria and facilitate elemental composition elucidation, also in retrospective analysis [52], but both can be affected by low ion abundance [53,54]. According to our data, mass accuracy and RIA are not reliable when ion abundance is lower than 10^4^ counts, which would correspond to 0.2 ng/mL PnTX-G in methanol in our conditions of analysis (see Supplementary Information). We assessed the isotopic pattern of the protonated ion [M + H]^+^ of PnTX-G in sample MUS120523 (694.4677 *m/z*; 5.9 ng/mL PnTX-G in the methanolic extract) and found that only the first isotope (C_41_^13^CH_64_NO_7_) was measured accurately (<10 ppm), thus the complexity of shellfish matrices negatively influenced the abundance threshold for isotopic pattern analysis. Therefore the used of this confirmation criteria was not feasible in our study.

Product ion scans from previously isolated precursor ions can provide reliable information for confirmation, but they cannot be used as a post-run screening tool [52], unless the precursor ions had been isolated in the ion trap analyzer. The LTQ Orbitrap Discovery^®^ can perform “data dependent MS^2^ experiments” (*i.e*., the precursor ion found in the full MS scan triggers an MS^2^ scan). However, the success of these experiments depend on the careful selection of an appropriate abundance threshold for the precursor ion in the full MS scan: too low thresholds lead to MS^2^ scans even in methanol blanks, while higher thresholds did not trigger any scan even in positive control samples. Thus, for the samples analyzed in this study, the levels of cyclic imines around few parts-per-billion did not allow performing data dependent MS^2^ experiments.

**Figure 3:**
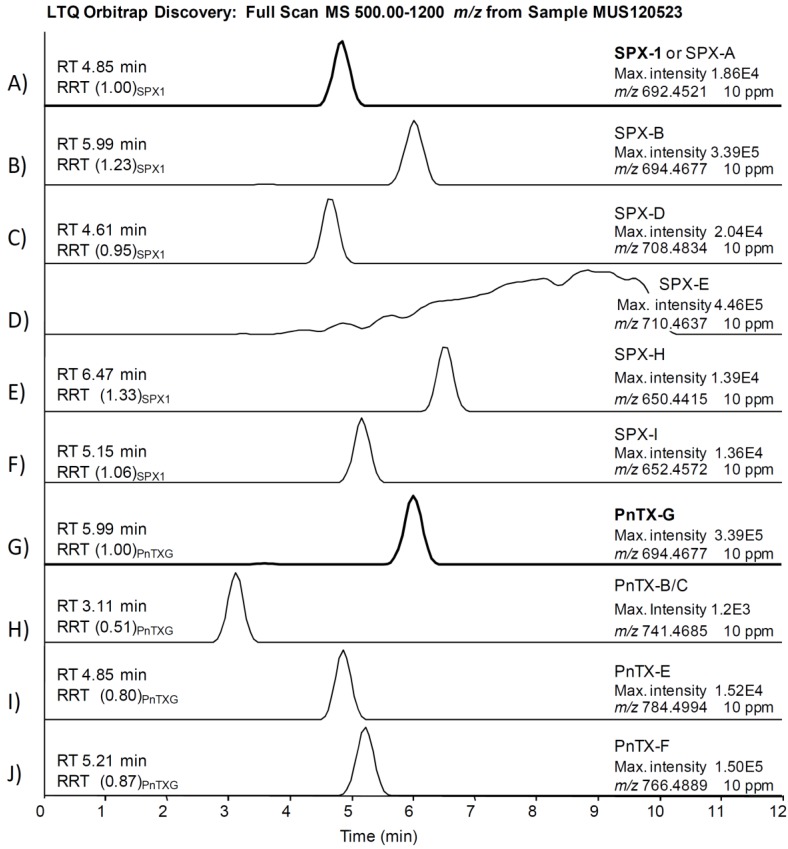
(**A**–**J**) Extracted chromatograms of precursor ions of spirolides and pinnatoxins listed in Table 1 that produced a peak with 10 ppm mass window tolerance in sample MUS120523. Retention times (RT) in minutes; RRT in brackets is the RT relative to the main toxin in the group (SPX-1 or PnTX-G).

**Figure 4:**
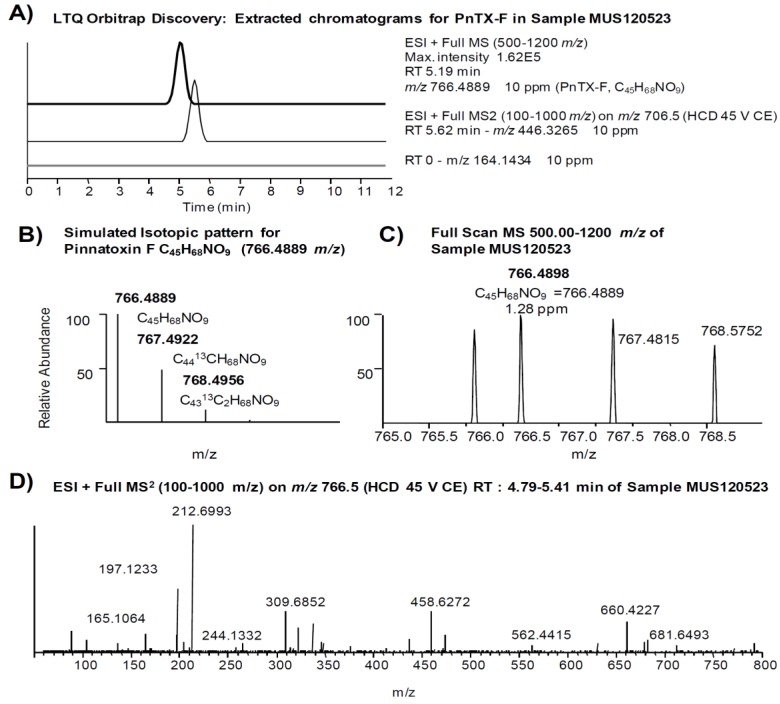
Study of a false positive of PnTX-F in sample MUS120523: (**A**) extracted ion chromatogram of the precursor ion (766.4889 *m/z*) in the MS scan, and an extracted chromatogram of the diagnostic fragments (164.1434 *m/z* and 446.3265 *m/z*) in the MS^2^ product scan (10 ppm mass window in both cases); (**B**,**C**) isotopic pattern of the precursor ion (simulated and found, respectively); and (**D**) MS^2^ product scan on the precursor ion (766.5 *m/z*).

The comprehensive study of sample MUS120523 illustrates our strategy applied to identify PnTXs and SPXs. We searched for all PnTXs and SPXs listed in Table 1 using a 10 ppm filter around the exact mass of the precursor ions. Figure 3 shows that 9 out of 22 precursor ions produced a chromatographic peak in the sample, although the ion abundances were too low to apply isotopic patterns and MS^2^ products as confirmation criteria. We focused on the peak produced by 766.4889 *m/z* (PnTX-F, Figure 3J), since the abundance of this ion was in the same order of magnitude as the ion abundance of the confirmed PnTX-G. Figure 4 illustrates the extracted ion chromatograms (Figure 4A), evaluation of the isotopic pattern (Figure 4B,C) and the MS^2^ full scan on 766.5 *m/z* (Figure 4D). We rejected the presence of PnTX-F in the sample, since: (i) the fragment 164.1434 *m/z* (the actual cyclic imine in the structure) was not present in the MS^2^ product ion scan scan (Figure 4A,D); (ii) the diagnostic fragment 446.3265 *m/z* was present but not at the same retention time as the precursor ion (Figure 4A); (iii) the isotopic pattern of the precursor ion did not correspond with the simulated isotopic pattern for PnTX-F formula: the isotopes A + 1 (C_44_^13^CH_68_NO_9_) and A + 2 (C_43_^13^C_2_H_68_NO_9_) were not present (with 10 ppm mass tolerance) and the isotopic ion ratios of the present ions did not fit the expected pattern (Figure 4B,C); (iv) the MS^2^ product ion scan from the precursor ion [M + H]^+^ of PnTX-F did not reveal any fragments that could have been related with toxins from the cyclic imine group (Figure 4D).

We confirmed that all peaks in Figure 3 (except peaks of PnTX-G and SPX-1, Figure 3A,G) were false positives. Before applying the confirmatory tests in HRMS in the rest of the samples, we screened them in the 3200QTrap^®^ using the diagnostic fragments listed in Table 1. Some samples showed peaks in MRM that could correspond to diagnostic fragments of PnTX-G and SPX-1 analogs, but they were proved to be false positives during the confirmatory analysis in HRMS. Therefore, according to our identification criteria, only PnTX-G and SPX-1 were present in the sample above the limits of detection.

### 2.3. Search for Acyl Ester Derivatives of Pinnatoxins and Spirolides

Acyl ester derivatives have been proven for toxin analogs belonging the three major types of cyclic imines (SPXs, PnTXs, GYMs) and described in shellfish samples from Norway, Canada and Tunisia [15,18,55]. Unlike SPXs and GYMs, PnTXs resist the alkaline hydrolysis protocol applied for the indirect determination of acyl ester analogs of the okadaic acid group of toxins. The presence of these shellfish metabolites can be evidenced by an increment in the concentration of free PnTXs after hydrolysis, compared to the concentration in the crude extract [18] However, acyl esters of SPXs cannot be assessed with this approach. We hydrolized the three mussel samples with the highest PnTX-G concentration to look for PnTX-G acyl derivates, but the concentration of PnTX-G only increased about 20%, much less than the 3-fold increase in PnTX-G concentration reported in Canadian shellfish [18]. Further investigation of the samples by precursor ion scans of the product 164.1 *m/z* in a 3200QTrap^®^ did not provide evidence on the presence of PnTXs or SPXs acyl esters in the samples (see Supplementary Information). Our results are in line with other studies that reported free PnTXs in oysters with no significant levels of acyl ester metabolites of PnTXs being present [17]. Thus, the metabolization of free toxins into acyl ester derivatives should always be studied in different shellfish species and/or in their physiological state.

### 2.4. Quality Assessment of the Methods

#### 2.4.1. Low Resolution MS with the Triple Quadrupole 3200QTrap®

Our method, developed on the triple quadrupole 3200QTrap^®^, provided the first hint of the presence of cyclic imines in samples of mussels, oysters and passive sampler devices from Catalonia (Spain). According to the Commission Decision 2002/657/EC [56], the confirmatory potential of the low resolution MS method is enough for PnTX-G and SPX-1 (one precursor ion, three product ions, ratio of abundance between product ions, and retention times). However, the method relying on the triple quadrupole mass analyzer constrained its applicability for the rest of toxin analogs lacking of reference standards. For them, the method was used as a screening tool in combination with high resolution MS as a confirmatory technique. The quantitative method presented comparable results for PnTX-G and SPX-1 (Table 4), showing good intra-day and inter-day precision (<3.1% RSDr and 7.1% RSDR, for SPX-1 in oysters), high sensitivity, good linearity, and small variation in retention time.

Matrix components might influence identification parameters, such as product ion ratio [57], but it was not the case in our study. Product ion ratios were very reproducible in the calibration curves prepared in methanol (<0.5% variation), and the influence of the matrices was completely negligible for PnTX-G ion ratios (<0.3% variation). Product ion ratios for SPX-1 where slightly different in spiked mussels compared to those in methanol (−1.0%), and especially in oysters (−4.9% variation), but these variations fell in the tolerance ranges proposed by the Commission Decision 2002/657/EC (±25% variation allowed) [56].

**Table 4:** Quality assessment of the quantification on the triple quadrupole 3200QTrap^®^ mass spectrometer (three days, single laboratory). [M + H]^+^ ions were 692 *m/z* and 694 *m/z* for SPX-1 and PnTX-G respectively. Product ions monitored were 674 *m/z*, 444 *m/z* and 164 *m/z* for SPX-1 (MRM1 as the quantifier, MRM2 and MRM3 as the qualifiers respectively); and 676 *m/z*, 458 *m/z* and 164 *m/z* for PnTX-G (MRM1, MRM2 and MRM3 respectively). Product ion ratios were calculated as the relative area (in percentage) of the qualifier MRM2 compared to the area of the quantifier MRM1. Variations in product ion ratios in mussels and oysters represent the difference (in percentage) between product ion rations measured in metanolic standard solutions and in spiked samples of mussels and oysters. LODs and LOQs in mussels were not trustable because the spiked sample was not completely blank. Later analysis on a PnTX-G free mussel sample had an LOD = 0.2 μg/kg and LOQ = 0.3 μg/kg (different methodology for limits calculation, see Results and Discussion and Experimental Section).

		SPX-1	PnTX-G
Linearity range (ng/mL) (*n* = 15)		2.5–50	2.5–50
RT (average, *n* = 75)		6.33	7.36
% drift		0.3	0.2
Product ion ratios (%) (*n* = 30)	Area MRM2/Area MRM1	40.7	77.1
	% variation in mussels	−1.0%	0.3%
	% variation in oysters	−4.9%	0.1%
Recovery (%) (*n* = 9)	Mussel	91.6	77.6
	Oyster	81.0	66.6
Intra-day precision (RSDr) (%) (*n* = 9)	Mussel	2.6	2.9
	Oyster	3.1	2.5
Inter-day precision (RSDR) (%) (*n* = 9)	Mussel	7.0	6.8
	Oyster	7.1	6.2
LOQ (μg/kg) (*n* = 3)	Mussel	19.6	56.1
	Oyster	0.7	0.9
LOD (μg/kg) (*n* = 3)	Mussel	5.5	10.8
	Oyster	1.1	0.5

The average recovery (Table 4) for SPX-1 was over 80% for mussels and oysters, but shellfish tissues may suppress the ion signal for PnTX-G quantification, since recoveries were below 80% (Table 4). We found negative matrix effects with mussels and oysters for GYM-A in a previous work [58], thus structurally related PnTX-G may suffer the same problem. We addressed this issue by correcting the quantification of the samples with recovery values obtained for the spiked samples.

Limits of detection and quantification were very low for both toxins in oysters (<1.5 µg/kg), but unexpectedly high (up to 56.1 μg/kg for PnTX-G) in mussels (Table 4). This can be explained because the mussel tissue used as a blank had trace levels (below LOQ) of SPX-1 and PnTX-G. Carryover did not cause this problem since the methanol blanks analyzed after the most concentrated level of the calibration curves never showed signal for these toxins. We could not find any mussel sample (from *n* = 12) with undetectable levels of PnTXs and SPXs at the time of the study. In 2014, we had access to a mussel sample collected in the same shellfish harvesting area with no traces of PnTXs. Since PnTX-G standard solution is not commercialized, its availability is extremely restricted. Thus, we could not repeat the experiments to calculate LOQ and LOD using the same methodology. Nevertheless, we spiked a metanolic extract of a mussel sample at 38 µg PnTX-G/kg mussel tissue and estimated the LODs and LOQs with five injections. The new LOD and LOQ would be 0.2 and 0.3 µg PnTX-G/kg mussel, a much lower result that would fit better with the S/N ratios we observed in samples and in standard solutions. For instance, the same spiking in methanol yielded to LODs and LOQs of 0.08 and 0.19 μg PnTX-G/L.

#### 2.4.2. High resolution MS with the LTQ Orbitrap Discovery®

The confirmatory method with the LTQ Orbitrap Discovery^®^ for PnTX-G and SPX-1 included the precursor and three product ions measured at high resolution (mass accuracy <2 ppm), in addition to one product ion ratio and retention times. Relative isotope abundances could not be applied to precursor ions due to lack of sensitivity.

High resolution MS reduces dependence on analytical standards for qualitative analysis. For instance, to confirm PnTXs and SPXs analogs without reference standards, the measurement of the precursor and three diagnostic product ions by HRMS would satisfy the guidelines of the Commission Decision 2002/657/EC [56].

Mass accuracy measurements for PnTX-G and SPX-1 were very reliable (<2 ppm in all cases, Table 5) for the precursor and product ions selected. Even for the lowest concentration analyzed of the standard solutions (2.5 ng/mL SPX-1 and 0.95 ng/mL PnTX-G), the mass accuracy was very good (<1 ppm). Thus, the method could reliably confirm the presence of SPX-1 and PnTX-G even at very low concentrations.

**Table 5:** Quality assessment of identification (LTQ Orbitrap Discovery^®^, mass resolution FTWM 30,000 at 400 *m/z*). RT: retention time. Averages of 13 injections in two days at five concentrations (2.5 to 40 ng SPX-1/mL; 0.95 to 190 ng PnTX-G /mL).

	NRC CRM SPX-1				NRC CRM PnTX-G			
RT (min)								
Average % drift	4.75 2				5.92 1			
	Fragment 1	Fragment 2	Fragment 3	Precursor	Fragment 1	Fragment 2	Fragment 3	Precursor
Exact mass	164.1434	444.3108	674.4415	692.4521	164.1434	458.3265	676.4572	694.4677
**Mass Accuracy (Δ ppm)**								
Average	−0.3	0.1	0.4	0.5	−0.4	−0.5	−0.1	0.1
Max.	−2.0	−1.6	−0.9	0.7	−1.7	−1.8	−1.4	−1.6
**Ion Ratios**								
% of Frag 1 intensity	100	53	50		94	69	100	
% difference		19	23		22	15		

Variations in retention times were below 2%, but the variation in product ion ratios was about 20% in the methanolic solutions of the standards (concentrations 2.5 to 40 ng/mL SPX-1 and 0.95 to 190 ng/mL PnTX-G, see Experimental Section). The use of product ion ratios for identification and confirmation has been questioned in several studies due to their lack of robustness, especially at low concentrations [59,60,61,62], which seem to concur with the lack of reproducibility in our product spectra acquired with the LTQ Orbitrap Discovery^®^.

## 3. Experimental Section

### 3.1. Standards and Chemicals

Certified reference standard solution of 13-desmethyl SPX-C was purchased from the Institute for Marine Bioscience of the National Research Council (NRC) (Halifax, NS, Canada) (SPX-1, 7.0 ± 0.4 µg/mL). Certified reference standard solution of PnTX-G was not commercially available, but a pre-released material (sealed ampoules not yet certified) was kindly donated by the NRC for the purpose of this study (non-certified concentration ~1.9 µg/mL).

For LC-MS/MS analysis in low resolution, we used acetonitrile (ACN) hypergrade for LC-MS, and methanol gradient grade for HPLC were purchased from Merck (Darmstadt, Germany). Ammonium hydroxide (28% in water; ≥99.99% trace metals basis), was purchased from Sigma-Aldrich (Steinheim, Germany). Ultrapure water was obtained through a Milli-Q purification system (resistivity >18 MW cm) from Millipore (Bedford, MA, USA). For high resolution LC-MS/MS, HPLC grade methanol, ultrapure water and acetonitrile were purchased from LAB-SCAN (Dublin, Ireland) and ammonium hydroxide (28% in water; ≥99.99% trace metals basis), was purchased from Sigma-Aldrich (Dublin, Ireland).

### 3.2. Preparation of Extracts

Blue mussels (*Mytilus galloprovincialis*) and Pacific oysters (*Crassostrea gigas*) were collected from the shellfish harvesting areas of Catalonia, Spain (NW Mediterranean Sea). A triple-step extraction with methanol (10 mL) was performed on whole homogenated tissues (1 g) according to the procedure proposed by Gerssen *et al.* [63] which was also validated intra-laboratory by our group [58]. Analytical balance Sartorius 1702 (Goettingen, Germany), a vortex-mixer MS2 Minishaker (IKA Labortechnik, Staufen, Germany) and a centrifuge Jouan MR 23i (Thermo Fisher Scientific Inc., Waltham, MA, USA) were used. Crude extracts were filtered through polytetrafluoroethylene (PTFE) 0.2 μm membrane syringe filters. The alkaline hydrolysis of the samples was performed according to the EURLMB SOP [64] based on the protocol developed by Mountfort *et al.* [65]. Solid-phase adsorption toxin tracking (SPATT) [66] devices used as passive samplers of dissolved lipophilic toxins in seawater were prepared and desorbed in methanol following the protocol explained in Caillaud *et al*. [67]. Briefly, the SPATT devices were prepared with ~10 g of wet adsorbent styrene-divinylbenzene resin (DIAON^®^ HP20, Mitsubishi Chemical Corporation, Tokyo, Japan) and remained immersed in the marine environment for a week. For toxin desorption, the resin was removed, washed with ultrapure water and filtered under vacuum. Then the resin was extracted twice with methanol 100% in a solvent:resin ratio of 8:1 mL/g of resin. Finally, the methanolic extracts were combined and evaporated, and the dry extract was dissolved in 5 mL of 100% methanol for LC-MS analyses.

### 3.3. Chromatographic Separation

Toxins were separated on a Waters X-Bridge™ C8 column (guard column 2.1 × 10 mm^2^, 3.5 μm particle size, column 2.1 × 50 mm^2^, 3.5 μm particle size; Waters, Milford, MA, USA). This analytical column was ethylene-bridged hybrid (BEH), designed to work on a wide range of pH between 1 and 12.

For low resolution LC-MS/MS, the chromatographic separation was performed on an Agilent 1200 LC system (Agilent Technologies, Santa Clara, CA, USA) consisting of a binary pump (G1312B), four channel degasser (G1379B), thermostated low carry-over autosampler (G1367C + G1330B), and column oven (G1316B). For high resolution LC-MS/MS, chromatography was performed with an Accela LC system (Thermo Fisher Scientific, Hemel Hempstead, UK). Alkaline mobile phases (pH 11) were prepared according to Gerssen *et al.* [63,68]: mobile phase A consisted of 6.7 mM of ammonia in ultrapure Milli-Q water; mobile phase B consisted of 6.7 mM of ammonia in 90/10 *v/v* ACN/Milli-Q water. Mobile phases were filtrated through 0.2 μm nylon-membrane. Chromatography of lipophilic toxins under alkaline pH was validated in out laboratory and provided better sensitivity for cyclic imines than other chromatographic conditions [58].

The column oven temperature was set at 30 °C and the flow rate was 0.5 mL/min. The elution gradient was optimized [58] and started at 20% mobile phase B (mpB), reached 100% mpB in 8 min, held for one minute, then back to 20% mpB in 0.5 min and equilibrated for 2.5 min before the next run started. The diverter valve was programed to deliver the eluent from column to waste for the first 1.5 min.

Injection volume was optimized at 10 μL in the Agilent LC system and 15 μL in the Accela LC system. The sample compartment was set at 4 °C. The needle was washed with methanol in the flush port of the autosampler before every injection to avoid carryover.

### 3.4. Mass Spectrometry

For low resolution LC-MS/MS, a hybrid triple quadrupole linear ion trap 3200QTrap^®^ mass spectrometer (MS) equipped with a TurboV™ electrospray ion source (Applied Biosystems, Foster City, CA, USA) operating at atmospheric pressure and positive ionization mode with the following parameters: curtain gas 20 psi, collision gas 4 (arbitrary units), ion spray voltage 5500 V, temperature 500 °C, nebulizer gas 50 psi, heater gas 50 psi, interface heater On. The MS was operated in the multiple reaction monitoring (MRM) mode. The precursor ions monitored [M + H]^+^ were 692 *m/z* and 694 *m/z* for SPX-1 and PnTX-G respectively. Product ions monitored were 674 *m/z*, 444 *m/z* and 164 *m/z* for SPX-1 (MRM1, MRM2 and MRM3, respectively); and 676 *m/z*, 458 *m/z* and 164 *m/z* for PnTX-G (MRM1, MRM2 and MRM3, respectively). MRM1 was the quantifier while MRM2 and MRM3 were qualifiers; these ions were selected according to their diagnostic power [8,16,17] and included the cyclic imine (MRM3). The precursor and product ions applied for the screening of analogs of PnTX-G and SPX-1 were the nominal *m/z* as those listed in Table 1. Resolution of the quadrupoles was set at unit and the rest of MS parameters were: dwell time 150 ms, declustering potential 86 V, entrance potential 7 V, collision cell entrance potential 30 V and collisions energy 45 eV. Analyst software v1.4.2 was used for the entire MS tune, instrument control, data acquisition and data analysis.

For high resolution LC-MS/MS, a hybrid high-resolution LTQ Orbitrap Discovery^®^ FT-MS (Thermo Fisher Scientific, Hemel Hempstead, UK) operating in positive ionization mode with a heated electrospray interface (HESI) was used. The ion source tune method values were optimized and set at: capillary temperature 250 °C, capillary voltage 47.5 V, tube lens 90 V, sheath gas 50 (arbitrary units), auxiliary gas 10 (arbitrary units). The analysis was divided into two experiments: First experiment was a full MS scan event from 100 *m/z* to 800 *m/z*; second experiment involved full MS^2^ scans from precursor ions previously isolated in the first-stage ion trap mass analyzer: 692 *m/z* (Isolation Width 4.0 Da) at 45.0% collision energy (CE) in the high collision dissociation (HCD) cell for SPX-1, and 694 *m/z* (Isolation Width 4.0 Da) with the same CE for PnTX-G. Resolution of 30,000 FWHM (at 400 *m/z*) was selected (the maximum provided by the instrument). To improve mass accuracy measurements, we used 453.34353 *m/z* as lock mass, a frequent and consistent background interference from the membrane nylon disks used for filtering the mobile phases [69,70]. The lock mass offers real time recalibration of the mass spectral region. The tuning, control, data acquisition and data analysis were done with Xcalibur^®^ software v2.0.7.

### 3.5. Method Performance and Quality Control

A quality assessment of the quantification capabilities of the method on the triple quadrupole 3200QTrap^®^ mass spectrometer was conducted. The single laboratory three days assessment relied on the concepts described in Taverniers *et al.* [71]; and the guidelines proposed by the Regulation (EC) 657/2002 [56] and the EU-Harmonized SOP for Lipophilic toxins [64]. The external standard calibration curves were prepared in methanol (LC-MS grade) from an initial multi-toxin stock solution of 100 ng/mL of NRC CRM 13-desmethyl SPX-C and NRC RM PnTX-G, with five levels in the range of 2.5 ng/mL to 50 ng/mL. Sensitivity of the method was evaluated as the slope of the calibration curves [72]. Linearity was assessed by two parameters: correlation coefficients of the quantification curves had to be greater than 0.98; and the deviation of the slopes between consecutive calibration curves had to be lower than 25%. The drift in the retention times (RT) corresponds to the variation of RT along the three days and was considered as acceptable below 3%. Product ion ratios were calculated as the relative area (in percentage) of the qualifier MRM2 compared to the area of the quantifier MRM1. Ion ratios were calculated in the external standards of the calibration curves, and variations in product ion ratios in mussels and oysters were measured as the difference (in percentage) between product ion rations measured in the external standards solutions and in spiked samples of mussels and oysters.

The recovery (ratio between quantified and spiked concentration in percentage, %R) and precision (intra-day relative standard deviation RSDr, inter-day RSDR) were calculated with blank homogenated samples of mussel and oyster, spiked at 50 μg/kg of NRC CRM SPX-1 and NRC RM PnTX-G. The spiked samples were analyzed in three consecutive days, three replicates per day, and quantified against the external standard calibration curves.

Limits of detection and quantification (LODs and LOQs) where calculated by the IUPAC method [73] considering the improves recommended in later studies [74]:

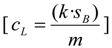
(1)
Where *c_L_* is the concentration that generates a signal equal to three times (*k* = 3 for LOD) or ten times (*k* = 10 for LOQ) the standard deviation of the noise in the blanks (*s_B_*), divided by the analytical sensitivity (*m*), which is the slope of a matrix matched calibration curve in the concentration range of the limits. The spiked mussel and oyster where diluted in methanol five times (1 to 25 μg/kg) to calculate *m*, and noise was calculated as the height of MRM2 for LODs and MRM1 for LOQs in the vicinity of RT of the toxins, with three injection replicates of the blank samples (not spiked). Average limits include one replicate per day (*n* = 3).

After the quality assessment, the spiked samples were considered as “internal material” and used to correct the quantification of the shellfish samples with its recovery. Samples were injected in duplicate and quantified against the external standard calibration curves (less squared adjustment of the linear regression using peak area).

### 3.6. Identification and Assessment of Identification Criteria

A two days single laboratory quality assessment of the identification criteria with a LTQ Orbitrap Discovery^®^ (mass resolution FTWM 30,000) was performed. This brief assessment only included the standards (five dilutions from 2.5 ng/mL to 40 ng/mL of SPX-1, 0.95 ng/mL to 190 ng/mL of PnTX-G), not the spiked samples. We evaluated the RT and its drift along two days. The mass accuracy (Δ, in ppm) of the precursor ions (measured in the full MS scan) and the selected fragments (in the full MS^2^ scan) was evaluated using the features provided by the qualitative browser of Xcalibur software.

Ion ratios and their variation (relative standard deviation, in percentage) in the MS^2^ spectra were calculated as the relative intensity of the fragment ions compared to the most intense one in the full MS^2^ scan.

Identification was confirmed when the precursor ion was found in the full MS scan and the main fragments (Table 1) were found in the full MS^2^ scan with a mass tolerance of 5 ppm. The retention times and product ion ratios compared to those obtained from reference standards were also used as confirmation criteria to unambiguously confirm the presence of SPX-1 and PnTX-G in the samples.

## 4. Conclusions

We unequivocally confirmed and quantified PnTX-G and SPX-1 in shellfish and passive samplers (SPATT devices) from Catalonia, Spain. This is the first report of pinnatoxins in Spain and the first time that spirolides have been detected in Catalonia. We developed and discussed a comprehensive strategy to avoid false positives of toxin analogs of PnTXs and SPXs by LC-MS/MS analysis, with applicability to other toxin groups and analogs, which consisted of:

(1) Fast and sensitive screening in low resolution MS with a 3200QTrap^®^ triple quadrupole using diagnostic transitions for all PnTXs and SPXs described up to date.
(2) Confirmation analysis in high resolution MS with an LTQ Orbitrap Discovery^®^ that included the inspection of isotopic patterns and MS^2^ spectra, and the look for the precursor ion and three diagnostic product ions with a mass tolerance window selected accordingly to the method performance.

Evidences of acyl ester metabolites of PnTXs could not be obtained from mussel samples, neither after application of the alkaline hydrolysis procedure nor through precursor ion scan mass spectrometric experiments.

Cyclic imines should be included in the shellfish safety monitoring programs of lipophilic marine toxins by LC-MS methods, even if they are not regulated, to better assess their presence in shellfish and favor exposure studies that would enable a reliable risk analysis for consumers. The introduction of benchtop high resolution mass spectrometry instruments in research and control laboratories may enhance the capabilities to ensure unequivocal confirmation of emerging marine toxins, as shown in this work. However, a proper strategy should be applied combining isotopic patterns, MS and MS^2^ accurate mass measurements, signal thresholds, and tolerances applied to mass filters in order to avoid potential false positives or negatives.

## Supplementary Files

Supplementary File 1 Supplementary Information (PDF, 417 KB)
