# Supplementary material for: Confirmation of Pinnatoxins and Spirolides in Shellfish and Passive Samplers from Catalonia (Spain) by Liquid Chromatography Coupled with Triple Quadrupole and High-Resolution Hybrid Tandem Mass Spectrometry
Source: Mar Drugs. 2014 Jun 23;12(6):3706–32. doi: 10.3390/md12063706 (PMC4071598; doi:10.3390/md12063706)
Supplement: Supplementary File 1 — Supplementary Information (PDF, 417 KB) [file marinedrugs-12-03706-s001.pdf]

Supplementary Information

Figure S1. (**A**–**C**): Three methanol blanks screened for spirolides (SPX-1 to SPX-I, see Table 1 in the main article) in the full MS scan at 10 ppm mass error tolerance. Blanks were included in a batch of sixty samples (shellfish and SPATT samples) every six samples. (**A**) First methanol blank; (**B**) Fifth methanol blank; (**C**) Tenth methanol blank. Carryover was discarded by checking the methanol blanks analyzed after the most concentrated level of the calibration curves, which never showed signal for PnTX-G of SPX-1 at their retention times. Peaks did not correlate with the expected relative retention time of the target analytes, but there are no standards available for PnTXs and SPXs other than PnTX-G and SPX-1.

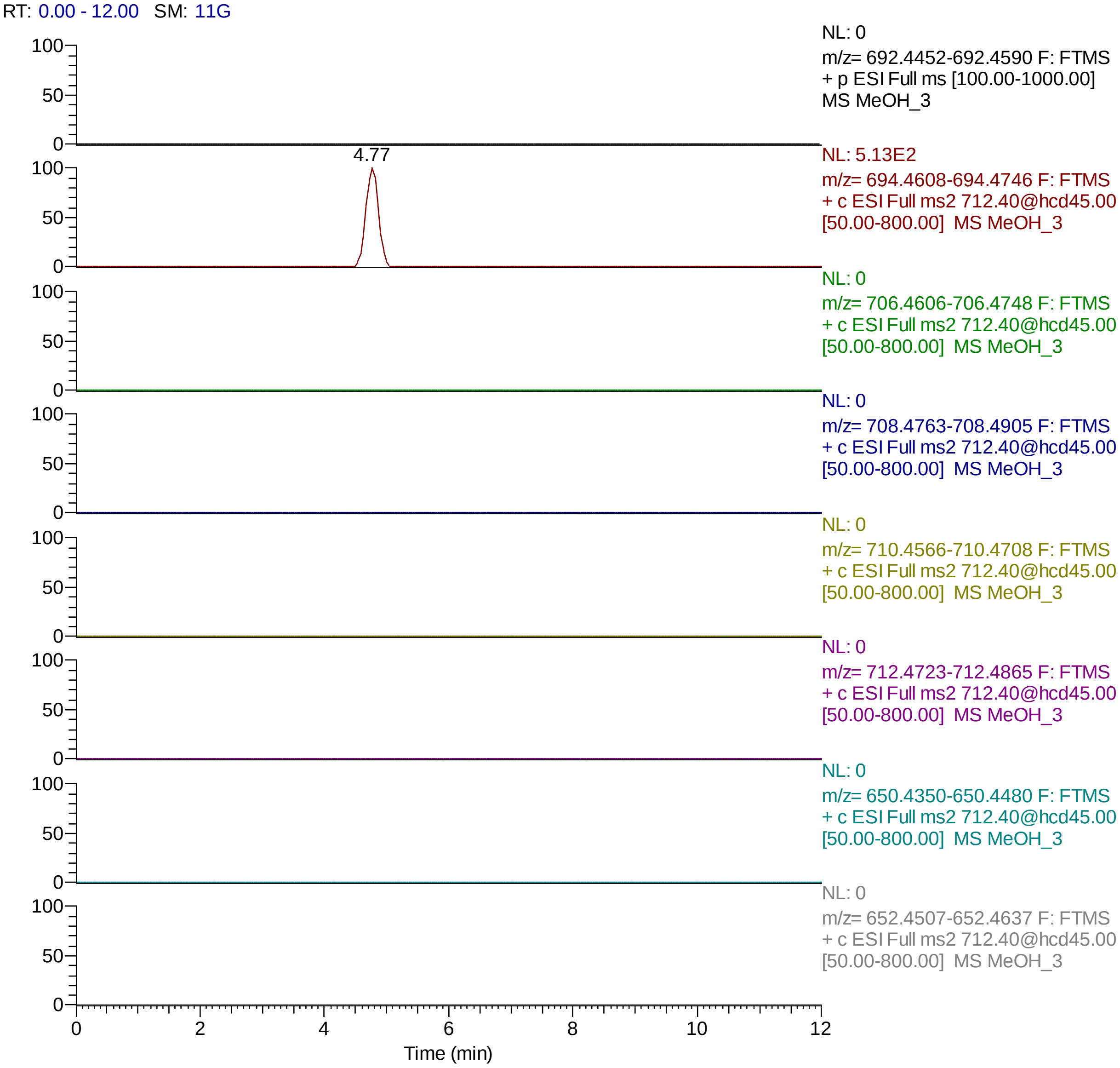

(**A**)

Figure S1. *Cont.*

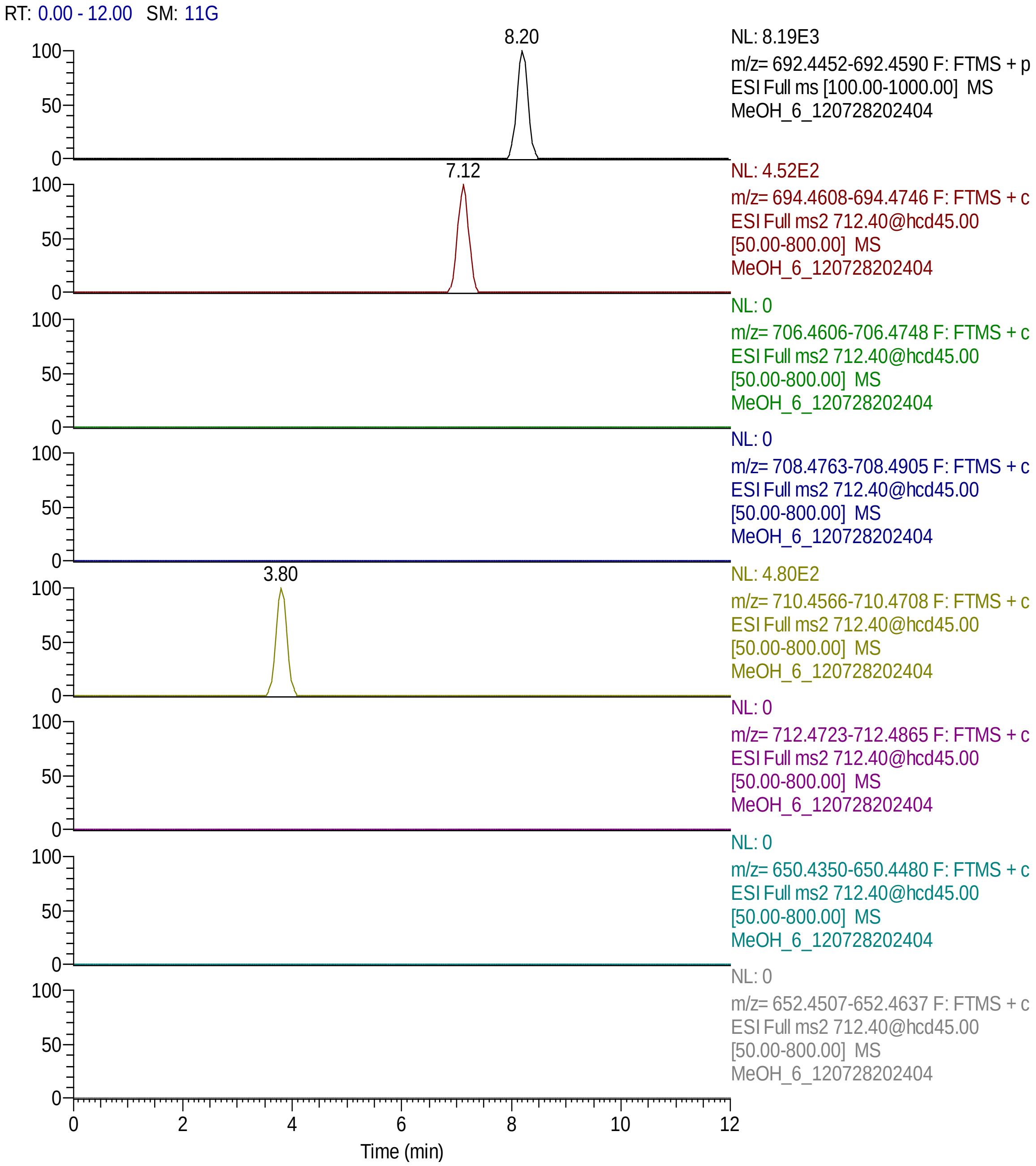

(B)

Figure S1. *Cont.*

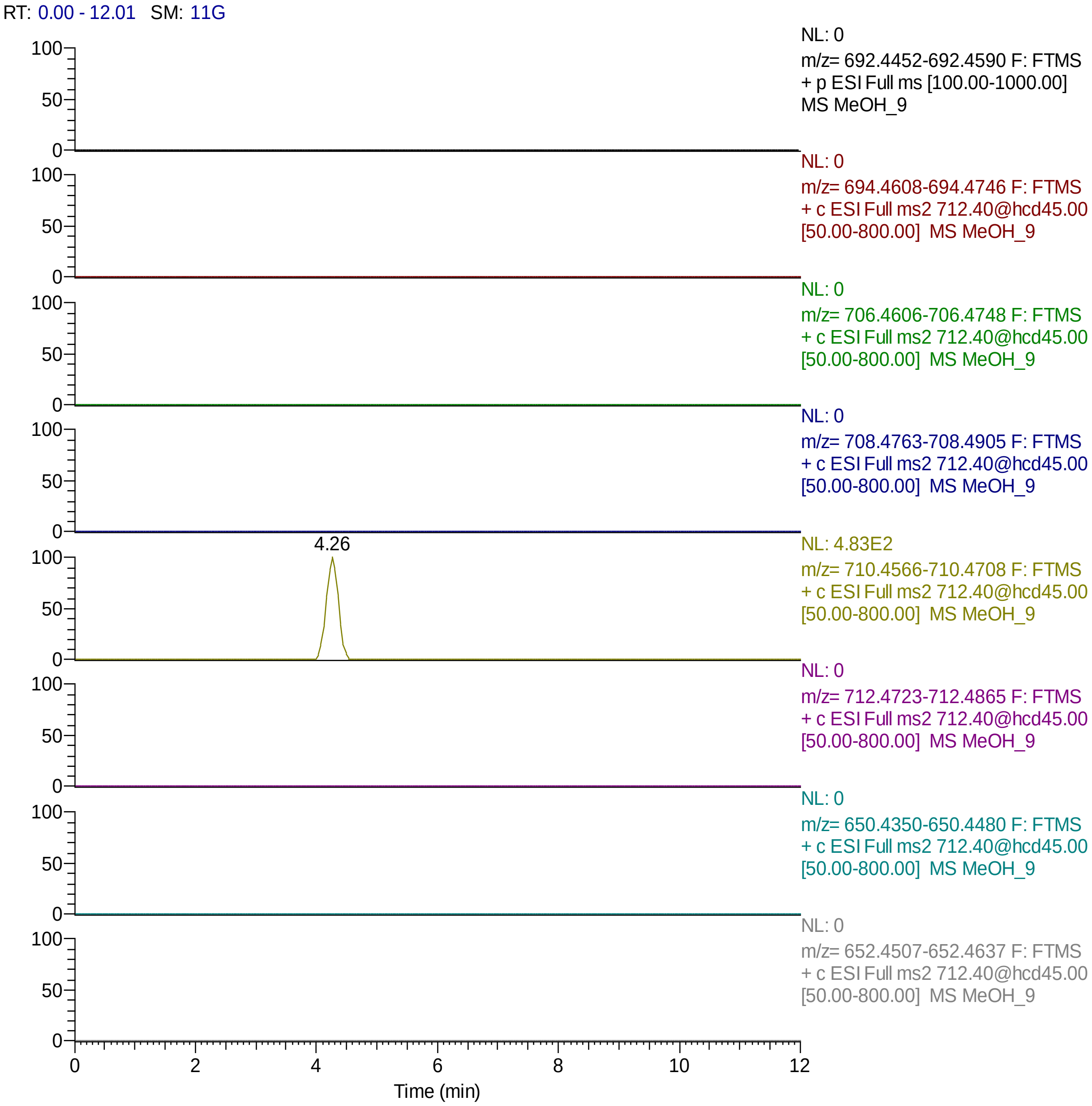

(C)

Figure S2. Isotopic pattern simulation for PnTXG ($C_{42}H_{64}NO_7$) calculated by Xcalibur® 2.07.

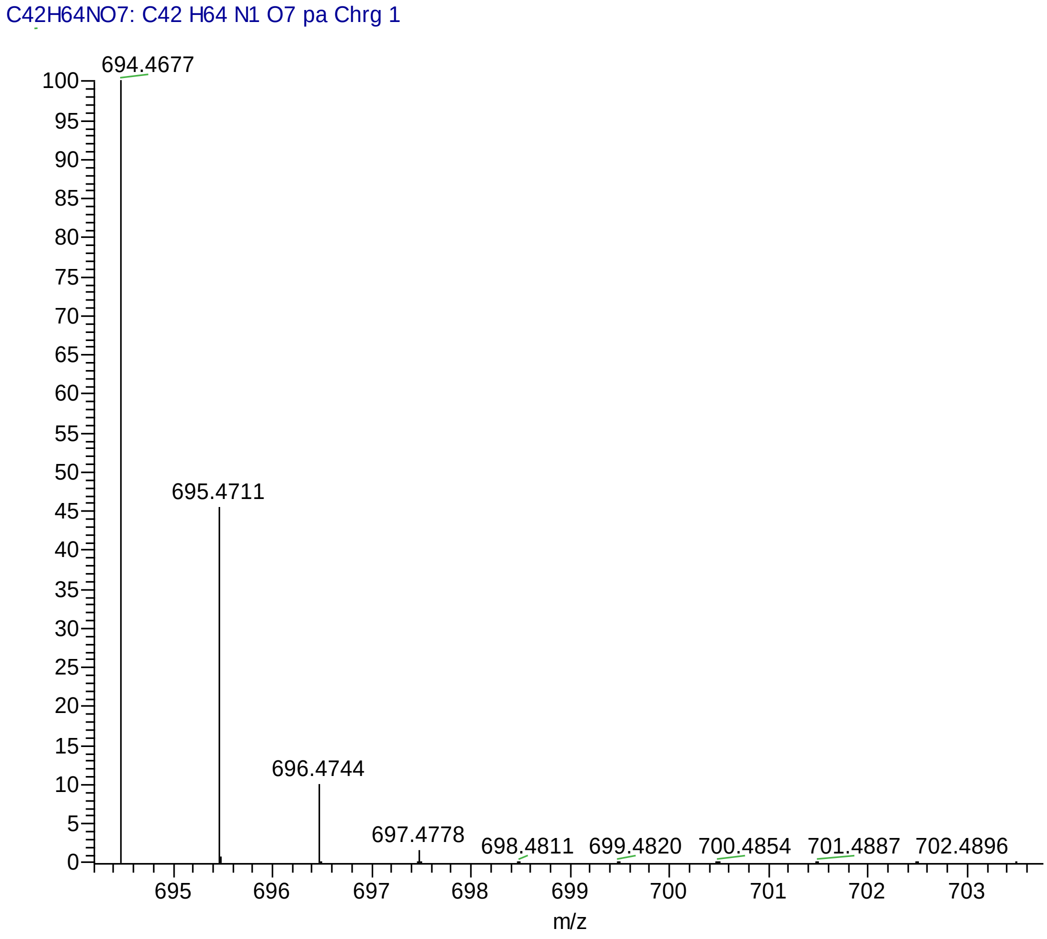

m/z	Intensity	Relative
694.4677	618847.0	100.00
695.4711	281117.8	45.43
696.4744	62330.1	10.07
697.4778	8988.6	1.45
696.4720	8902.1	1.44
695.4740	4555.2	0.74
697.4753	4043.9	0.65
695.4648	2285.8	0.37
696.4774	2069.3	0.33
695.4719	1650.1	0.27
696.4681	1038.3	0.17
698.4811	947.9	0.15
698.4787	896.6	0.14
696.4753	749.6	0.12
697.4807	458.8	0.07
697.4715	230.2	0.04
697.4787	166.2	0.03
699.4820	129.3	0.02
699.4845	77.9	0.01
698.4841	66.2	0.01

Figure S3. Contour Plotof LC-MS precursor scan for *m/z*164.2 (CE 45 V) with a Triple Quadrupole QTRAP 3200®. Sample of mussel from Alfacs Bay (May 2012) with 60 μg/kg free PnTXG. The characteristic pattern with diagonal lines is evident in the figure, which could be consistent with an acylation of PnTXG with fatty acids; although PnTXG esters described in literature [1] were formed by longer chains with *m/z* values between *m/z* 904 (C14:0) and *m/z* 1034. (C24:5). The low concentration of these compounds constrained their characterization by MS^2.

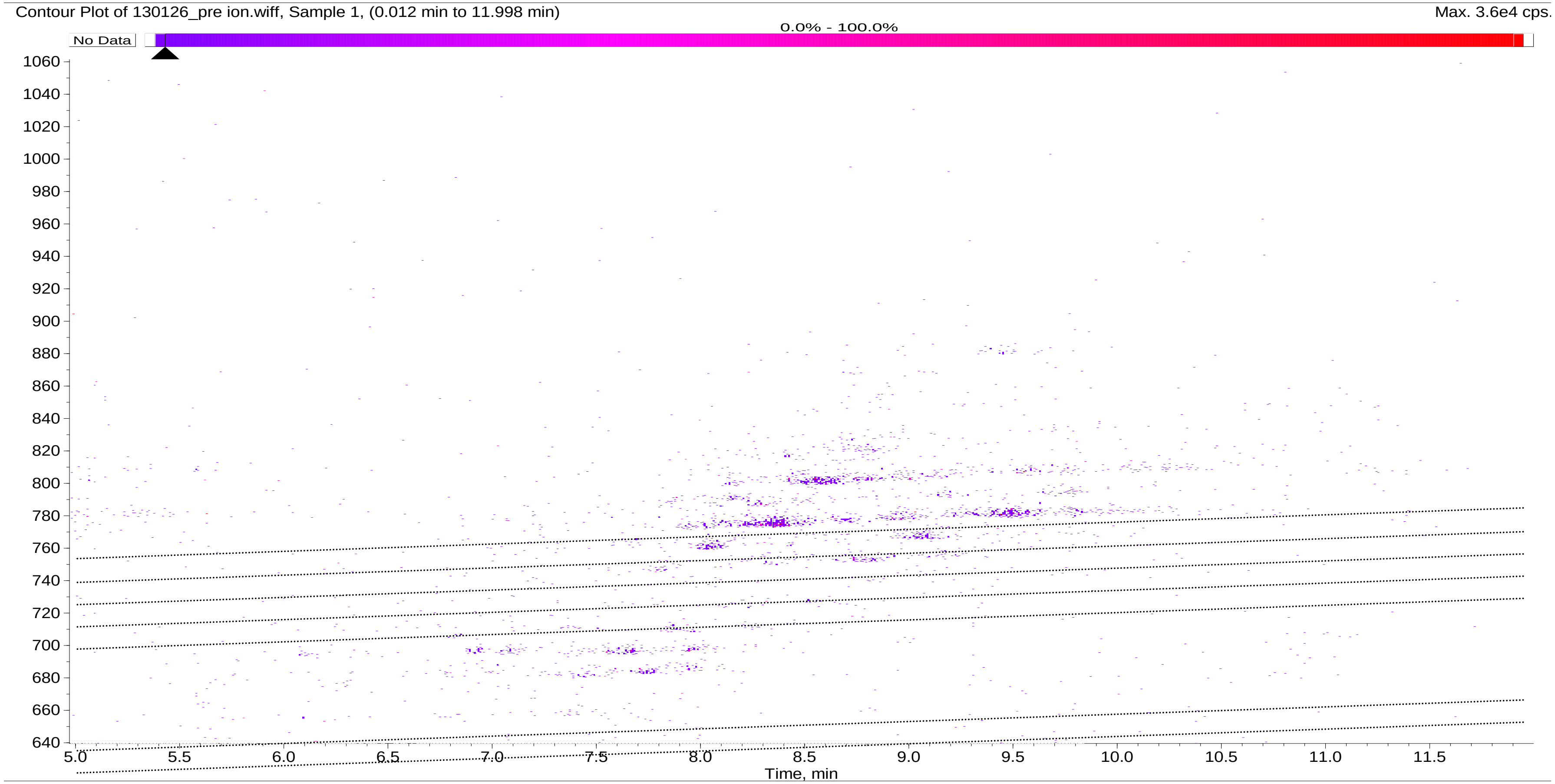

Table S1. Assigned mass errors (Δ in ppm) to the molecular formula and theoretical *m/z*, and ion intensities on the precursor ion (A) and the first three isotopes (A + m) of PnTX-G (n = 2 for 19 and 1.9 ng/mL; n = 1 for 190 and 0.2 ng/mL). Mass errors larger than 5 ppm are in bold, also the intensities associated with the analysis that yielded these high mass errors. When intensity were below 1E4, mass accuracy dropped below 5 ppm.

**Isotopes—PnTX-G (*m/z*)**		**Concentration—PnTX-G (ng/mL)**			
		**190**	**19**	**1.9**	**0.2**
		**Δ (ppm)**			
A (Prec ion) $C_{42}H_{64}NO_7$	(*m/z* 694.4677)	−0.1	−0.4	−0.1	0.4
A + 1 $C_{42}{}^{13}CH_{64}NO_7$	(*m/z* 695.4711)	−0.1	−0.5	−1.2	−20.6
A + 2 $C_{42}{}^{13}C_2H_{64}NO_7$	(*m/z* 696.4744)	0.1	−0.1	−1.4	−22.7
A + 3 $C_{42}{}^{13}C_3H_{64}NO_7$	(*m/z* 697.4778)	0.7	1.1	−50.5	15.2
**Intensity Counts**					
A (Prec ion) $C_{42}H_{64}NO_7$	(*m/z* 694.4677)	1.56×10^7	1.95×10^6	2.17×10^5	1.74×10^4
A + 1 $C_{42}{}^{13}CH_{64}NO_7$	(*m/z* 695.4711)	7.24×10^6	8.88×10^5	1.01×10^5	9.36×10^3
A + 2 $C_{42}{}^{13}C_2H_{64}NO_7$	(*m/z* 696.4744)	1.80×10^6	2.18×10^5	1.57×10^4	8.10×10^2
A + 3 $C_{42}{}^{13}C_3H_{64}NO_7$	(*m/z* 697.4778)	3.13×10^5	2.81×10^4	6.74×10^3	7.24×10^2

Table S2. Differences in (%) in intensity ratios between the isotopes (A + m) and the precursor ion (A) compared to the simulated isotopic pattern for PnTX-G (n = 2 for 19 and 1.9 ng/mL; n = 1 for 190 and 0.2 ng/mL). For low concentrations (1.9 and 0.2 ng/mL PnTX-G) the difference between the simulated isotopic ratios and the measured isotopic ratios is very evident (over 35%).

**Isotopes–PnTXG *(m/z)***	**Concentration—PnTX-G (ng/mL)**			
	**190**	**19**	**1.9**	**0.2**
A + 1/A (Prec ion)	−2	0	−2	−19
A + 2/A (Prec ion)	−15	−11	38	54
A + 3/A (Prec ion)	−39	0	−175	−187

Table S3. Isotopic pattern analysis in sample MUS120523. Assigned mass errors (in ppm) to the molecular formula and theoretical *m/z*, and ion intensities on the precursor ion (A) and the first three isotopes (A + m) of PnTX-G. Differences in (%) in intensity ratios between the isotopes (A + m) and the precursor ion (A) compared to the simulated isotopic pattern for PnTX-G. Only A + 1 could be measured with appropriate mass accuracy, and its relative intensity compared to the precursor ion (A) similar to the simulated relative intensity berween A + 1 and A (12.5% difference). A + 2 could not be measured accurately and A + 3 was not found.

**Isotopes—PnTXG (*m/z*)**		**Sample MUS120523**			
		***m/z* Found**	**Intensity Counts**	**Mass Error (ppm)**	**% Diff. Int. Ratios**
A (Prec ion) $C_{42}H_{64}NO_7$	(*m/z* 694.4677)	694.4669	1.63×10^5	1.15	
A + 1 $C_{42}{}^{13}CH_{64}NO_7$	(*m/z* 695.4711)	695.4702	6.46×10^4	1.29	12.5
A + 2 $C_{42}{}^{13}C_2H_{64}NO_7$	(*m/z* 696.4744)	696.4883	2.11×10^4	−19.96	−28.7
A + 3 $C_{42}{}^{13}C_3H_{64}NO_7$	(*m/z* 697.4778)	Not found	Not found	Not found	Not found

Reference

1. McCarron, P.; Rourke, W.; Hardstaff, W.; Pooley, B.; Quilliam, M. Identification of pinnatoxins and discovery of their fatty acid ester metabolites in mussels (*Mytilus edulis*) from eastern Canada. *J. Agric. Food Chem.* **2012**, *60*, 1437–1446.

© 2014 by the authors; licensee MDPI, Basel, Switzerland. This article is an open access article distributed under the terms and conditions of the Creative Commons Attribution license (http://creativecommons.org/licenses/by/3.0/).
